# Enhancing the use of sodium-glucose cotransporter-2 inhibitors in type-2 diabetic patients with chronic kidney disease, through a key performance indicator program

**DOI:** 10.5339/qmj.2025.50

**Published:** 2025-06-30

**Authors:** Muhammad Asim, Ramzi Abdul Rahiman, Muhammad Abdul Azim Baig, Hassan Al-Malki

**Affiliations:** 1 Department of Medicine, Hamad General Hospital, Doha, Qatar; 2 Hamad Medical Corporation, Doha, Qatar *Email: masim@hamad.qa

**Keywords:** SGLT2 inhibitors, type-2 diabetes mellitus, chronic kidney disease, quality improvement, key performance indicator, prescribing behavior

## Abstract

**Background:**

Despite substantial clinical evidence and recommendations from international societies supporting the use of sodium-glucose cotransporter-2 inhibitors (SGLT2i) for managing patients with type-2 diabetes (T2D) and chronic kidney disease (CKD), their adoption has remained limited. To address this, the nephrology quality improvement (QI) team at Hamad Medical Corporation (HMC), Qatar, implemented a key performance indicator (KPI) program in March 2022, aiming to ensure that at least 80% of eligible T2D-CKD patients at HMC were receiving SGLT2i by December 31, 2023.

**Methods:**

The use of SGLT2i and angiotensin-converting enzyme inhibitor (ACEi) or angiotensin II receptor blocker (ARB) in T2D-CKD patients attending nephrology clinics at HMC hospitals were assessed through retrospective surveys using a cluster-based sampling approach. Nephrology physicians were then updated on the results of these retrospective surveys as well as evidence from the SGLT2i trial and guidelines. The aims, objectives, and targets of the KPI program were clearly defined. Three additional surveys were conducted at 6-month intervals. A multifaceted QI intervention approach—combining audit, feedback, leadership engagement, and peer consultation—was implemented to drive improvement.

**Results:**

Retrospective surveys conducted in November 2021 and February 2022 revealed that 38% and 44% of eligible T2D-CKD patients were receiving SGLT2i therapy, compared to 98% and 99% for ACEi/ARB. Four months after implementing the KPI program, the July 2022 survey revealed no change in SGLT2i use (41%), while ACEi/ARB prescription rates remained near 100%. Following QI interventions in November 2022, the February 2023 survey revealed a significant increase in SGLT2i use, rising to 88%, with SGLT2i initiation in naive patients increasing from 34% to 61%. The final survey conducted in August 2023 showed that 84% of patients were receiving SGLT2i therapy.

**Conclusion:**

Our KPI program boosted SGLT2i prescription rates for eligible T2D-CKD patients in nephrology clinics, resulting in a 47% rise from 41% to 88%. It overcame prescription inertia and accelerated the guideline adoption by combining real-time feedback, leadership engagement, and peer discussions. The sharp rise in new prescriptions following November 2022 feedback underscores its direct influence on behavior modification rather than a broader trend.

## BACKGROUND

The beneficial effects of sodium-glucose cotransporter-2 inhibitors (SGLT2i) on renal and cardiovascular outcomes have been consistently supported by randomized controlled trials in patients with type-2 diabetes (T2D).^1–3^ Hence, the Kidney Disease: Improving Global Outcomes (KDIGO) 2022 Clinical Practice Guidelines, along with a consensus report from the American Diabetes Association (ADA) and KDIGO in 2022, provided clear guidance for the implementation of care strategies aimed at enhancing clinical outcomes for patients with T2D and chronic kidney disease (CKD).^4,5^ These groups recommend an angiotensin-converting enzyme inhibitor (ACEi) or angiotensin II receptor blocker (ARB) for patients with diabetes, hypertension, and albuminuria, titrated to the maximum antihypertensive or highest tolerated dose; and a SGLT2i with proven kidney or cardiovascular benefit for T2D-CKD patients, and estimated glomerular filtration rate (eGFR) >20 ml/minute/1.73 m^2^. Despite the evidence and the strong guideline endorsements, the adoption of SGLT2i in managing T2D-CKD patients has been limited. International data show that SGLT2i prescription rates remain suboptimal in clinical practice. A study from China reported 12.3% of T2D-CKD patients were on SGLT2 inhibitors, while another study found that only 11.9% of American diabetes patients who met clinical criteria for SGLT2i therapy were prescribed the medication.^6,7^

Hamad Medical Corporation (HMC) is a network of hospitals that provides public healthcare in Qatar, offering subsidized services to citizens and residents.^8^ The nephrology quality improvement (QI) team at HMC operates a key performance indicator (KPI) program that focuses on setting measurable targets that are aligned with best practices and clinical guidelines. By tracking these indicators and regularly providing feedback to healthcare providers, the program motivates improvements in clinical practice. Given the observed gap between international practice and optimal care for T2D-CKD patients, the QI team implemented a KPI program in March 2022 to assess the utilization of SGLT2i for T2D-CKD patients at HMC and to ensure that at least 80% of eligible T2D-CKD patients at HMC were receiving SGLT2i by December 31, 2023.

## PATIENTS AND METHODS

The KPI program was approved, and the QI team was granted access to the records of nephrology patients by the Head of the Nephrology Section, in accordance with local policies for internal QI projects. Since the study was a QI project intended to enhance system processes, it was exempt from IRB approval.

To assess the baseline utilization of SGLT2i and ACEi/ARB in T2D-CKD patients at HMC, two retrospective surveys were conducted among patients who attended general nephrology clinics in November 2021 and February 2022. A cluster-based sampling approach was used, with general nephrology clinics across hospitals serving as sampling clusters. Clinics were sequentially selected at random, and all eligible patients with T2D-CKD within each selected clinic were included. This process continued until a total of 100 patients were reached in each survey. To achieve this and enhance representativeness, at least 75% of the nephrology clinics across all hospitals in HMC were included in the clusters. Clinical, laboratory, and prescription data were extracted from electronic medical records by the QI team. The collected data were used to compare SGLT2i and ACEi/ARB prescribing patterns. The data were entered into password-protected MS Excel sheets on secured Nephrology office computers. Access to data was restricted to delegated QI team members.

Diabetic kidney disease was clinically diagnosed with the presence of albuminuria, and/or reduced eGFR. CKD was defined as eGFR <60 ml/minute/1.73 m^2^ (regardless of albuminuria) or urine albumin creatinine ratio (ACR) ≥30 mg/g (≥3 mg/mmol) (regardless of eGFR). The eGFR was derived from the serum creatinine level using the CKD-Epidemiology Collaboration equation. If ACR was not available, the protein creatinine ratio was converted to its approximate ACR equivalent using the Johns Hopkins University School of Medicine calculator.^9^

From nephrology point of view, all adult T2D-CKD patients were considered eligible if the physician recorded the diagnosis of ‘T2D with CKD’/’diabetic CKD’/‘diabetic kidney disease’/’diabetic nephropathy’, and eGFR was >30 ml/minute/1.73 m^2^ (the eGFR limit for the use of SGLT2i was lowered to >20 ml/minute/1.73 m^2^ in 2023 to reflect new evidence of benefits and safety). Patients were excluded if they had a documented adverse effect or a contraindication to the use of SGLT2 inhibitors. Patients with polycystic kidney disease and those on renal replacement therapy (including kidney transplant) were also excluded.

All nephrology physicians were updated on the results of the retrospective surveys, briefed on the latest evidence and ADA/KDIGO guidelines advocating the use of SGLT2 inhibitors in adult T2D-CKD patients, and the aims and objectives of the KPI program. Following this, three additional 6-monthly audits and performance reviews were conducted to monitor the prevalence of SGLT2i and ACEi/ARB, as well as SGLT2i prescription rates among SGLT2i-naive patients with T2D-CKD in nephrology clinics. An independent physician verified the results by selecting sample data from all surveys. Chi-square and p-value were used to compare differences in prevalence and prescription rates before and after QI interventions.

A multifaceted QI intervention approach, combining education, audits with feedback, leadership engagement, and peer consultation, was used. Survey results were discussed with the whole nephrology team via Microsoft Teams and face-to-face group meetings, chaired by the Head of Nephrology Section. During these meetings, prescribing practices and gaps were highlighted. Feedback was also provided through group emails, enabling physicians to assess their performance against targets.

## RESULTS

**Figure 1:**
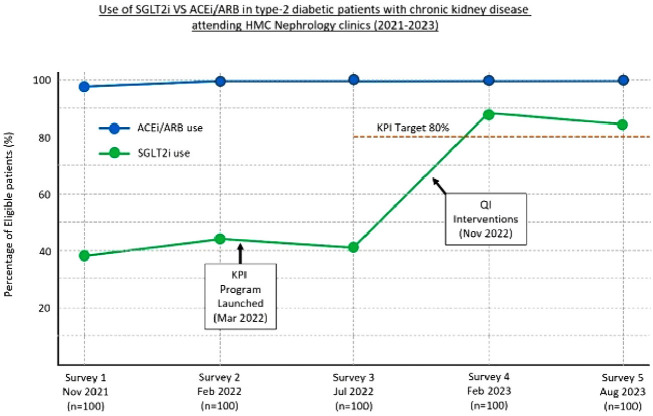
Percentage of eligible type-2 diabetic patients with CKD receiving SGLT2i therapy VS ACEi/ARB in five surveys.

The results are depicted in Figure 1. Retrospective surveys conducted among patients who attended nephrology clinics in November 2021 and February 2022 revealed that 38% and 44% of eligible T2D-CKD patients, respectively, were receiving therapy with SGLT2i. Conversely, ACEi/ARB were prescribed for 98% and 99% of these patients. Four months after the implementation of the KPI program, the July 2022 survey revealed no change in the use of SGLT2i. These agents were used in only 41% of eligible patients, while ACEi/ARB prescription rates approached 100%.

**Figure 2:**
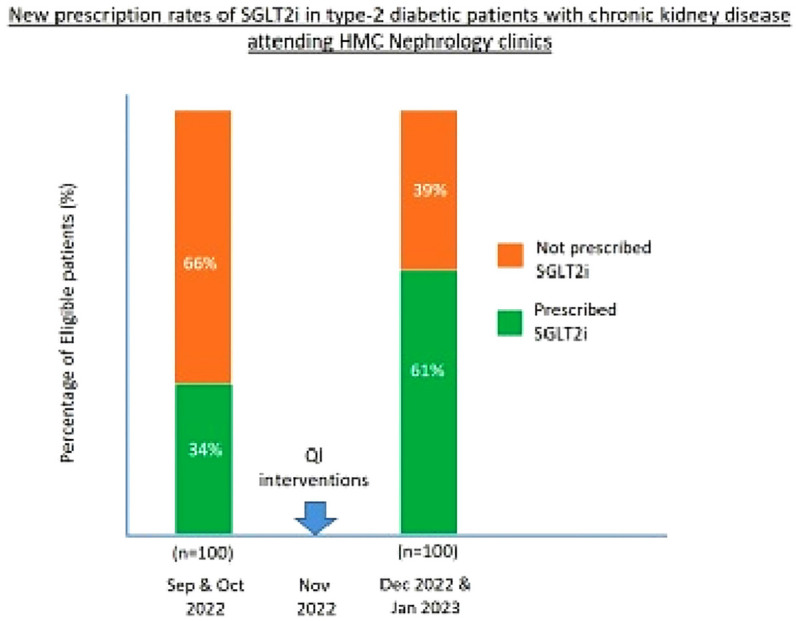
Comparison of new prescription rates of SGLT2i in eligible type-2 diabetic patients with CKD attending nephrology clinics before and after QI interventions.

Following QI interventions in November 2022, the follow-up survey in February 2023 indicated that the use of SGLT2 inhibitors had increased from 41% to 88% among eligible patients (as evidenced by a Chi-square statistic of 48.24 (df = 1) and a p-value <0.001). SGLT2i initiation rates in SGLT2i-naive T2D-CKD patients attending nephrology clinics increased from 34% to 61%, implying a 27% absolute increase in the prescribing rate (Figure 2). The final survey in August 2023 showed that the prevalence rate of patients receiving SGLT2 therapy was 84% (Figure 1).

## DISCUSSION

Our KPI program aimed to increase the proportion of T2D-CKD patients receiving SGLT2i therapy in nephrology clinics, given the compelling evidence of their efficacy in reducing CKD progression and cardiovascular risks, as well as the strong (level 1A) recommendations from KDIGO and ADA guidelines.^4,5^ Retrospective surveys conducted in November 2021 and February 2022 at HMC hospitals revealed that these agents were used in approximately 40% of eligible patients (Figure 1). In comparison, ACEi/ARB prescription rates approached 100%, likely due to clinicians’ familiarity with these agents and decades of evidence of their efficacy in slowing CKD progression and providing cardiovascular benefits.^10,11^ Based on the highly endorsed recommendations from the KDIGO and ADA guidelines, the KPI target for SGLT2i utilization was set at 80% of eligible patients by December 31, 2023.

Although clinicians were updated on the latest evidence supporting the use of SGLT2i and the results from the first two surveys, the July 2022 survey revealed no improvement in SGLT2i utilization, with the medication prescribed to only 41% of eligible patients (Figure 1). Conversely, ACEi/ARB continued to be universally prescribed.

This disparity indicated inertia in prescribing behavior, reflecting the tendency of healthcare providers to continue with established prescribing habits rather than adopting new evidence-based guidelines.^12^

The QI interventions implemented in November 2022 proved to be a watershed. Even though the surveys employed cluster sampling, the feedback was disseminated to all physicians. KPI data was shared through group meetings and emails, with the meetings chaired by the Head of the Nephrology Section. Real-time feedback provided a clear picture of gaps, reinforcing the need for improvement. Active leadership engagement drove performance and accountability, while peer discussions nurtured a supportive learning environment that reduced hesitation and boosted confidence in prescribing new drugs. The survey conducted in February 2023 indicated that the use of SGLT2i had increased from 41% to 88% among eligible patients (Figure 1). This observed change in proportions was highly significant, statistically. The next survey, conducted in August 2023, confirmed a sustained improvement in SGLT2i prescriptions, with 84% of patients receiving the medication, meeting the KPI target.

Since CKD patients often have additional comorbidities, SGLT2i are frequently prescribed by other specialties, particularly endocrinologists and cardiologists. To evaluate the impact of QI interventions on nephrologists, we compared the new prescription rates of SGLT2i among 100 SGLT2i-naive T2D-CKD patients attending nephrology clinics before and after the November 2022 feedback. We found that the new prescription rates increased from 34% to 61%, implying a 27% absolute increase (Figure 2). A Chi-square test of independence confirmed a statistically significant association between the QI interventions and the prescribing rates. A comparative analysis of SGLT2i use among T2D-CKD patients pre- and post-QI interventions, as well as the percentage of new initiators of SGLT2i, suggests that the increase in SGLT2i use was attributable to the KPI program rather than a natural increase over time.

Non-adherence to medical guidelines is a significant issue that can result in inconsistent care and poor patient outcomes. The KDIGO 2012 guidelines classify CKD risk using eGFR and urine ACR. A study analyzed patient characteristics and outcomes based on KDIGO classification using data from multiple databases. Higher albuminuria was associated with an increased prevalence of comorbidities and worse outcomes. Despite its importance, only 8.6% of 123,807 patients had urine ACR estimation.^13^ Another retrospective study evaluated physician adherence to ADA guidelines for the management of T2D. Results showed that only 32% received appropriate antidiabetic medication. Glycated hemoglobin targets were unmet in 53% of patients, and adherence to lipid and hypertension management was low at 43% and 66% respectively.^14^ Adherence becomes even more challenging when new medications, such as SGLT2i, are introduced. Key barriers to their adoption include low physician awareness, lack of confidence in prescribing, and clinical inertia.^7,15,16^ Hence, peer influence plays a crucial role—physicians are more likely to adopt new treatments when their peers do, particularly in shared practices or specialty groups.^17,18^

While consensus guidelines aim to improve healthcare delivery, their impact is limited without active implementation.^19,20^, We believe that the multifaceted approach, which combines audit, feedback, leadership engagement, and peer consultation, has significantly contributed to the success of our KPI program. Research supports the effectiveness of such multidimensional strategies in improving guideline uptake.^21–23^

Before generalizing these findings, it is important to consider potential limitations, including sample size and sampling methods. We used sequential random selection of clusters with full patient inclusion per cluster rather than a fully random cluster selection. Since cluster selection was not predetermined and instead ceased once the required sample size was met, this method may have introduced selection bias by favoring clinics selected earlier in the sequence. However, this approach ensured a broad representation of clinics. The uptake of a newly approved medicine is also influenced by pharmaceutical marketing, drug costs, healthcare infrastructure, insurance coverage, and reimbursement policies that may vary in settings outside of Qatar.

## CONCLUSION

Our KPI program significantly increased SGLT2i prescription rates for eligible T2D-CKD patients in nephrology clinics, increasing utilization from 41% to 88%, by integrating real-time feedback, leadership engagement, and peer discussions. This targeted approach addressed prescribing inertia and drove adherence to evidence-based guidelines. The observed increase in new prescriptions following the November 2022 feedback suggests that the KPI program played a pivotal role in changing prescribing behaviors rather than a general trend toward increased SGLT2i use over time. Future research could explore the long-term sustainability of these improvements and their impact on patient outcomes.

## List of abbreviations

**Table:** 

ACEi	Angiotensin-converting enzyme inhibitor
ACR	Albumin creatinine ratio
ADA	American Diabetes Association
ARB	Angiotensin II receptor blocker
CKD	Chronic kidney disease
CKD-EPI	Chronic Kidney Disease-Epidemiology Collaboration
eGFR	Estimated glomerular filtration rate
HMC	Hamad Medical Corporation
KDIGO	Kidney Disease: Improving Global Outcomes
KPI	Key performance indicator
PCR	Protein creatinine ratio
QI	Quality improvement
SGLT2i	Sodium-glucose cotransporter-2 inhibitors
T2D	Type-2 diabetes

## Conflicts of interest

None.
